# Investigation of Intercellular Salicylic Acid Accumulation during Compatible and Incompatible *Arabidopsis-Pseudomonas syringae* Interactions Using a Fast Neutron-Generated Mutant Allele of *EDS5* Identified by Genetic Mapping and Whole-Genome Sequencing

**DOI:** 10.1371/journal.pone.0088608

**Published:** 2014-03-04

**Authors:** Jessie L. Carviel, Daniel C. Wilson, Marisa Isaacs, Philip Carella, Vasile Catana, Brian Golding, Elizabeth A. Weretilnyk, Robin K. Cameron

**Affiliations:** McMaster University, Department of Biology, Hamilton, Ontario, Canada; University of Texas, United States of America

## Abstract

A whole-genome sequencing technique developed to identify fast neutron-induced deletion mutations revealed that *iap1-1* is a new allele of *EDS5* (*eds5-5*). RPS2-AvrRpt2-initiated effector-triggered immunity (ETI) was compromised in *iap1-1/eds5-5* with respect to *in planta* bacterial levels and the hypersensitive response, while intra- and intercellular free salicylic acid (SA) accumulation was greatly reduced, suggesting that SA contributes as both an intracellular signaling molecule and an antimicrobial agent in the intercellular space during ETI. During the compatible interaction between wild-type Col-0 and virulent *Pseudomonas syringae* pv. *tomato* (*Pst*), little intercellular free SA accumulated, which led to the hypothesis that *Pst* suppresses intercellular SA accumulation. When Col-0 was inoculated with a coronatine-deficient strain of *Pst*, high levels of intercellular SA accumulation were observed, suggesting that *Pst* suppresses intercellular SA accumulation using its phytotoxin coronatine. This work suggests that accumulation of SA in the intercellular space is an important component of basal/PAMP-triggered immunity as well as ETI to pathogens that colonize the intercellular space.

## Introduction

In response to pathogens *Arabidopsis* relies on various induced defenses. Basal resistance or PTI (PAMP-Triggered Immunity) is a defense response elicited by the recognition of conserved pathogen- or microbe-associated molecular patterns (PAMPs or MAMPs) by pattern recognition receptors [1]. Some bacteria, for example, certain pathovars of *Pseudomonas syringae*, are able to suppress PTI by delivering effector proteins into plant cells using a type-three secretion system [1]. Several of these effectors interfere with various stages of PTI, contributing to the pathogen's ability to cause disease on host plants [2]–[6]. In response, many plants possess resistance genes (R genes) that recognize effectors either directly or indirectly, leading to R gene-mediated resistance or Effector-Triggered Immunity (ETI) [7]. Some of the defense mechanisms associated with ETI are thought to overlap with those of PTI although they seem to occur more rapidly and with greater strength during ETI [1]. These defenses include extensive transcriptional reprogramming resulting in cellular changes such as expression of defense genes (e.g., *PR [PATHOGENESIS-RELATED]* genes), production of phytoalexins, salicylic acid (SA) biosynthesis, and cell-wall modifications [8]–[10]. In addition, ETI is often associated with the hypersensitive response (HR), a form of programmed cell death thought to contribute to inhibition of pathogen spread [11].

Many R genes encode NB-LRR (nucleotide-binding leucine-rich repeat) proteins which can be subdivided into two groups; those possessing N-terminal coiled-coil domains (CC-NB-LRR) and those with N-terminal TIR (Toll and Interleukin-1 receptor) domains (TIR-NB-LRR) [7]. CC- and TIR- type R genes are generally thought to function using separate pathways defined by their requirement for either *NDR1 (NON-RACE-SPECIFIC DISEASE RESISTANCE1)* or *EDS1 (ENHANCED DISEASE SUSCEPTIBILITY1)* respectively [12]. For example, *RPS2* encodes a CC-NB-LRR resistance protein that confers resistance to strains of *P. syringae* expressing *avrRpt2* [13], [14]. Similarly, the TIR-NB-LRR resistance protein RPS4 confers resistance to strains of *P. syringae* expressing *avrRps4* [15], [16].

Another form of induced disease resistance is the developmentally regulated Age-Related Resistance (ARR) response (reviewed in [17], [18]). In *Arabidopsis*, ARR results in enhanced resistance to certain pathogens with increasing plant age [19]. Specifically, 6-week-old Col-0 plants grown in short days limit the growth of *P. syringae* pv. *tomato (Pst)* to levels that are 10- to 100-fold lower than in 3-week-old plants. Unlike PTI and ETI, the molecular mechanisms underpinning ARR in *Arabidopsis* are only beginning to be understood.

A common player in many disease resistance pathways is the phytohormone SA (reviewed in [20], [21]). Wild-type *Arabidopsis* accumulates SA in response to inoculation with both virulent and avirulent *Pst* [22]. The importance of SA accumulation for different disease resistance responses is typically tested using transgenic and mutant plants with a reduced ability to accumulate SA. *NahG* plants expressing a bacterial salicylate hydroxylase gene convert SA to catechol and consequently accumulate very little SA [23]. *ICS1/SID2 (ISOCHORISMATE SYNTHASE1/SALICYLIC ACID INDUCTION DEFICIENT2)* encodes a key enzyme in the biosynthetic pathway responsible for most pathogen-responsive SA production in *Arabidopsis* [24], [25]. *EDS5/SID1 (ENHANCED DISEASE SUSCEPTIBILITY5)* encodes a multidrug and toxin extrusion (MATE) family protein that localizes to the chloroplast envelope and transports SA from its site of synthesis into the cytoplasm [26]–[29]. Both *sid2* and *eds5/sid1* mutants accumulate little SA in response to pathogens [22]. *NahG*, *sid2*, and *eds5* support higher growth of virulent strains of *P. syringae* compared to wild-type plants suggesting that SA accumulation is important in limiting pathogen growth even in a compatible (susceptible) interaction [22], [23], [30], [31]. ETI is compromised in *NahG* [8], [23], [32]–[34], *sid2*, and *eds5* [22], [32], [35] when initiated by several R genes (*RPS2*, *RPS4*, *RPM1*) interacting with their corresponding effectors. In experiments using type-three secretion system mutants or PAMPs (flg22) to initiate PTI in wild-type Col-0 or *sid2*, Tsuda et al. [36] demonstrated that SA accumulation is required for a successful PTI response. Thus, in *Arabidopsis*, SA accumulation is important in numerous ETI and PTI pathways.

SA accumulation is also required for the *Arabidopsis* ARR response to *Pst* as demonstrated by the ARR-defective phenotypes of *NahG*, *sid2-1*, and *sid1*/*eds5-3* [19], [37]. Examination of mature plants responding to *Pst* revealed 6-fold higher SA levels in intercellular washing fluids (IWFs) relative to young plants [38]. Anti-microbial activity was often observed in the IWFs of mature plants inoculated with *Pst* as demonstrated by inhibition of *in vitro Pst* growth [38]. Preventing SA accumulation in the intercellular space by pressure-infiltrating ARR-competent plants with salicylate hydroxylase disrupted their ability to undergo ARR. Conversely, adding exogenous SA to the intercellular space rescued ARR-defective mutants and enhanced ARR in wild-type Col-0 [38]. Taken together this data led to the hypothesis that SA may act as an antimicrobial agent in the intercellular space during ARR.

A classical mutant screen for mature plants with defects in ARR was used to identify genes involved in the ARR response, including *iap1-1 (important for the ARR pathway1-1)*. Along with their ARR-defective phenotype, mature *iap1-1* plants accumulate little SA [37]. In this work, a combination of genetic mapping, whole-genome sequencing, and complementation analysis was used to identify *iap1-1* as a mutant allele of *EDS5*. While mapping the *iap1-1* mutation, we investigated the role of IAP1 in RPS2- and RPS4-mediated ETI by measuring bacterial levels and monitoring HR cell death using trypan staining and electrolyte leakage. Intercellular SA accumulation is important during ARR and requires functional IAP1, therefore we investigated both inter- and intracellular SA accumulation during ETI (incompatible interaction) and during a compatible interaction with virulent *Pst* in young Col-0 and *iap1-1*. Our results suggest that inter- and intracellular SA accumulation is important during both compatible and incompatible interactions.

## Results

### The *iap1-1* mutant is partially compromised in resistance to *Pst(avrRpt2)* and *Pst(avrRps4)*

**Figure 1:**
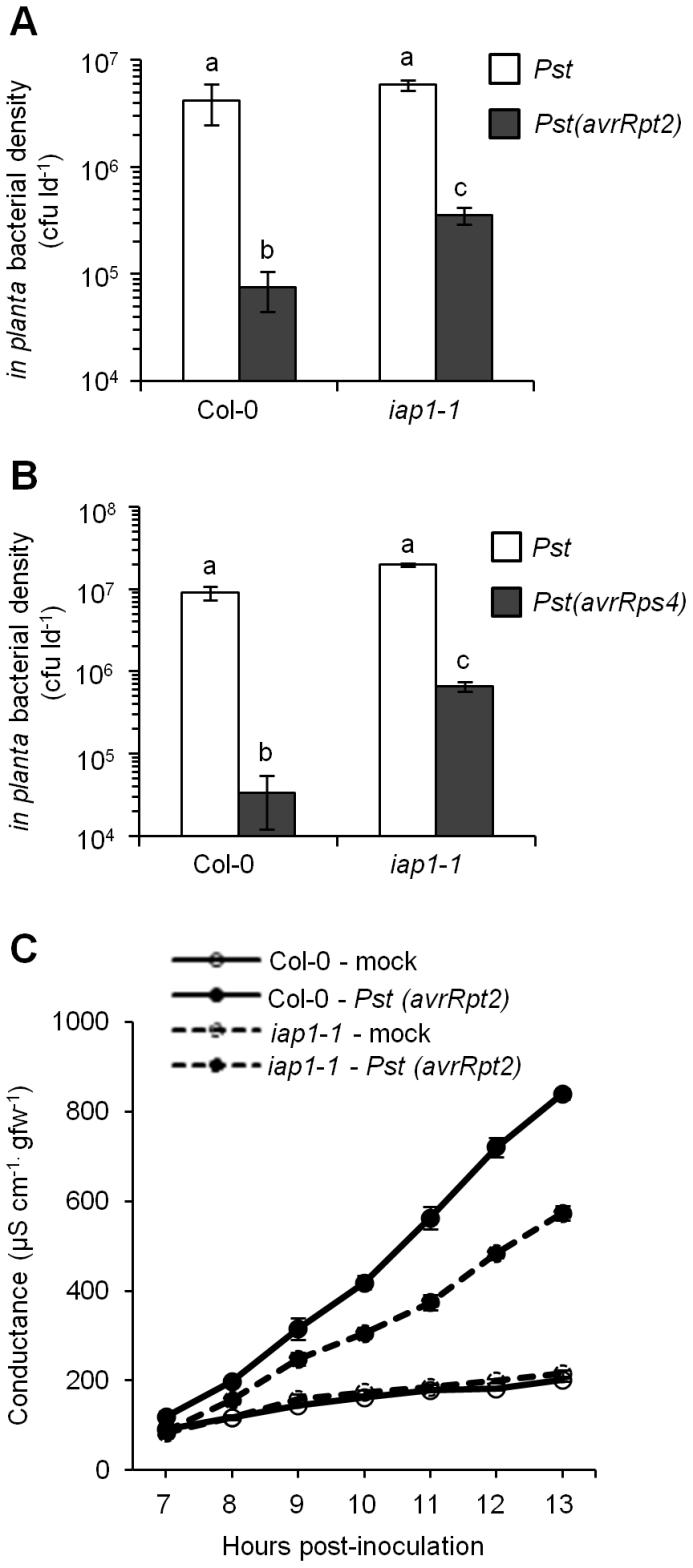
Effector-triggered immunity and the hypersensitive response in *iap1-1*. Three-week-old Col-0 and *iap1-1* were inoculated with 10^6^ cfu ml^−1^ *Pseudomonas syringae* pv. *tomato (Pst)* and *Pst(avrRpt2)* (A) or *Pst(avrRps4)* (B). *In planta* bacterial levels (colony-forming units per leaf disc [cfu ld^−1^]) were determined at 3 days post-inoculation and are presented as the mean ± standard deviation of three sample replicates. Different letters indicate significant differences (ANOVA, Duncan's MRT, *P*<0.05). Both experiments were repeated at least three times with similar results. (C) Electrolyte leakage was monitored in tissue collected from plants inoculated with 10^7^ cfu ml^−1^ *Pst(avrRpt2)* or 10 mM MgCl_2_ (mock) and is presented as the mean ± standard deviation of three samples. This experiment was repeated twice with similar results.

The *iap1-1* mutant is defective for ARR to virulent *Pst* [37]. To determine whether *IAP1* is also required during NDR1- or EDS1-dependent ETI/incompatible interactions, young plants at 3 weeks post-germination (wpg) were inoculated with 10^6^ cfu (colony-forming units) ml^−1^ *Pst*, *Pst(avrRpt2)*, or *Pst(avrRps4)* and *in planta* bacterial density was measured 3 days post-inoculation (dpi). *Pst(avrRpt2)* grew to significantly lower levels than *Pst* in both Col-0 and *iap1-1* indicating that an ETI response occurred (Figure 1A). However, *Pst(avrRpt2)* levels were significantly higher in *iap1-1* compared to Col-0 indicating that NDR1-dependent ETI to *Pst*(a*vrRpt2*) is partially compromised by the *iap1-1* mutation. Similar results were obtained when Col-0 and *iap1-1* were inoculated with *Pst(avrRps4)* which indicates that EDS1-dependent ETI to *Pst(avrRps4)* is also partially compromised by the *iap1-1* mutation (Figure 1B). Therefore, *IAP1* is required for a full and robust ETI response to *Pst* carrying effectors recognized by two distinct classes of resistance proteins.

### The hypersensitive response is partially compromised in *iap1-1*

Since the hypersensitive response (HR) is a common component of ETI elicited by both AvrRpt2 and AvrRps4 effectors [13], [14], [16] we investigated whether the *iap1-1* ETI defect was accompanied by a reduced HR. Four-week-old Col-0 and *iap1-1* were inoculated with 10^7^ cfu ml^−1^ *Pst(avrRpt2)* or 10 mM MgCl_2_ (mock-inoculated) and leaves were collected at 24 hours post-inoculation (hpi) and stained with trypan blue. Trypan blue does not pass through intact cell membranes of live cells therefore it selectively stains dying or dead cells and can be used to measure HR-associated cell death [47]. Visual analysis revealed little staining in mock-inoculated leaves whereas intense staining was observed in leaves inoculated with *Pst(avrRpt2)* (Figure S1). There was no obvious difference in the intensity of staining between Col-0 and *iap1-1* leaves suggesting that *iap1-1* undergoes a wild-type HR. By floating treated leaf tissue in a solution and measuring conductance, electrolyte leakage from dead or damaged cells can be quantified and used to track the progression of HR cell death over time [48]. Electrolyte leakage was monitored in tissue collected from 4-week-old Col-0 and *iap1-1* that were either mock-inoculated or inoculated with 10^7^ cfu ml^−1^ *Pst(avrRpt2)* (Figure 1C). Mock-inoculated leaves showed little change in electrolyte leakage over time. Electrolyte leakage from leaves inoculated with *Pst(avrRpt2)* increased substantially between 7 and 13 hpi. By 13 hpi, electrolyte leakage from Col-0 leaves inoculated with *Pst(avrRpt2)* was approximately 4-fold higher than mock-inoculated tissue, while the increase in electrolyte leakage from *iap1-1* leaves was more modest (2.7-fold increase compared to mock-inoculated tissue). Electrolyte leakage from *iap1-1* leaves was significantly less than from wild-type leaves between 10 and 13 hpi with *Pst(avrRpt2)* (T-test *P*<0.01) suggesting that HR-mediated cell death is compromised in *iap1-1*.

### Intracellular SA accumulation in response to *Pst* and *Pst(avrRpt2)*

**Figure 2:**
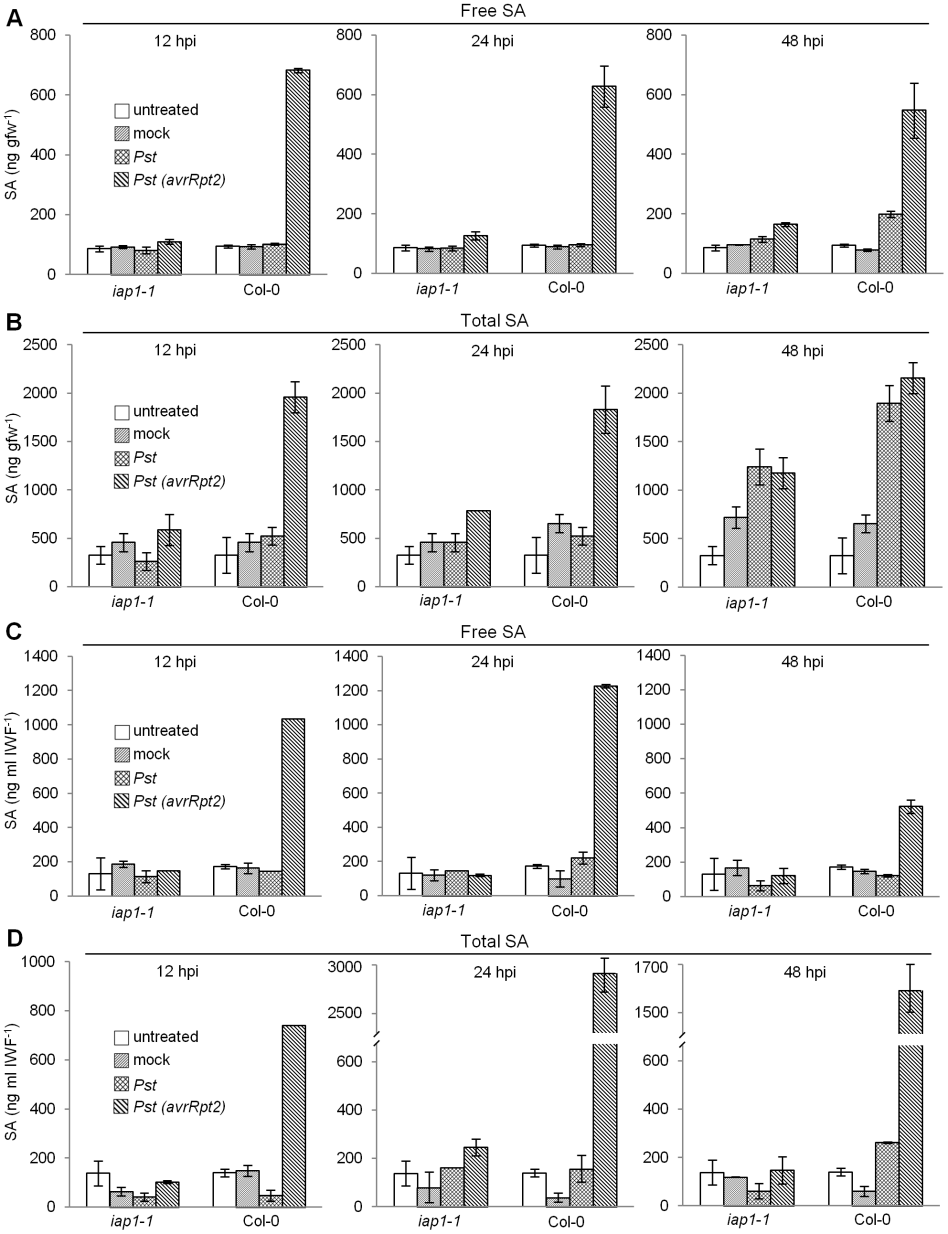
Salicylic acid accumulation in response to *Pst* and *Pst(avrRpt2)*. Four-week-old Col-0 and *iap1-1* were left untreated, mock-inoculated, or inoculated with 10^6^ cfu ml^−1^ *Pseudomonas syringae* pv. *tomato (Pst)* or *Pst(avrRpt2)*. Levels of intracellular free salicylic acid (SA) (A) and total SA (free SA+SA glucosides) (B) were determined in leaves from which intercellular washing fluids (IWFs) had been removed. Intercellular levels of free SA (C) and total SA (D) were determined using IWFs collected from the same leaves as in (A) and (B). The mean ± standard deviation of three replicate samples is shown. T-tests were performed to test for significant differences between means (see results section). This experiment was repeated twice with similar results.

SA is required for RPS2- and RPS4-mediated immunity [8], [22], [23], [32]–[34]. If young *iap1-1* plants accumulate little SA like mature *iap1-1* [37], this could explain the compromised ETI response observed in young *iap1-1*. To test this hypothesis, an ADPWH_*lux* SA biosensor [42] was used to measure SA levels in both intercellular (IWFs) and intracellular (leaves minus IWFs) compartments of young (4 wpg) Col-0 and *iap1-1* plants that were untreated, mock-inoculated, or inoculated with 10^6^ cfu ml^−1^ *Pst* or *Pst(avrRpt2)* (Figure 2). Intra- and intercellular SA results are discussed in this section and the next section respectively. Col-0 and *iap1-1* had similar levels of intracellular free SA in both untreated and mock-inoculated tissues at 12, 24, and 48 hpi (<100 ng gfw^−1^) (Figure 2A). Col-0 inoculated with *Pst* accumulated 199 ng gfw^−1^ intracellular free SA by 48 hpi, significantly more than mock-inoculated controls (T-test *P*<0.01), whereas intracellular free SA levels in *iap1-1* were similar to mock-inoculated controls at all time points. In response to *Pst(avrRpt2)*, Col-0 accumulated 546–682 ng gfw^−1^ intracellular free SA at 12, 24, and 48 hpi, whereas *iap1-1* accumulated very little intracellular free SA (109–164 ng gfw^−1^). This suggests that *iap1-1* accumulates little intracellular free SA in response to *Pst* or *Pst(avrRpt2)*. In addition, Col-0 accumulated higher levels of intracellular free SA in response to *Pst(avrRpt2)* compared to *Pst* (T-test *P*<0.01). Similar results were observed for intracellular total SA (free SA+SA-glucosides) with the exception that *iap1-1* accumulated high levels of total SA at 48 hpi with both *Pst* and *Pst(avrRpt2)*(Figure 2B).

### Intercellular SA accumulation in response to *Pst* and *Pst(avrRpt2)*

Intercellular SA accumulation is essential for ARR to *Pst*, and intercellular SA addition and subtraction experiments suggest that SA acts as an anti-microbial agent in the intercellular space during ARR [38]. To determine if SA accumulates in a similar manner in young plants undergoing ETI, SA levels were measured in IWFs collected from leaf tissue of young (4 wpg) Col-0 and *iap1-1* that were untreated, mock-inoculated, or inoculated with 10^6^ cfu ml^−1^ *Pst* or *Pst(avrRpt2)* (Figure 2C,D). IWFs from untreated and mock-inoculated Col-0 and *iap1-1* had similar levels of intercellular free SA at 12, 24, and 48 hpi (<200 ng ml IWF^−1^). Intercellular free SA levels in IWFs collected from Col-0 inoculated with *Pst* were similar to mock-inoculated controls. This is consistent with previous findings that young Col-0 accumulates little intercellular SA in response to *Pst* [38]. IWFs collected from *iap1-1* plants inoculated with *Pst* contained similar free SA levels relative to mock-inoculated controls. IWFs from Col-0 inoculated with *Pst(avrRpt2)* contained high levels of free SA at 12, 24, and 48 hpi (522–1226 ng ml IWF^−1^), whereas IWFs from *iap1-1* inoculated with *Pst(avrRpt2)* had similar levels as mock-inoculated controls (<200 ng ml IWF^−1^). Similar results were observed for total SA levels in IWFs with the exception that Col-0 accumulated a modest level of intercellular total SA in response to *Pst* at 48 hpi (262 ng ml IWF^−1^ compared to 60 ng ml IWF^−1^ in mock-treated plants, T-test *P*<0.01). Therefore, *iap1-1* accumulated little intercellular SA in response to *Pst* or *Pst(avrRpt2)*. In addition, wild-type Col-0 accumulated high levels of intercellular SA in response to *Pst(avrRpt2)*, and modest levels in response to virulent *Pst*.

### SA accumulation in response to *Pst(avrRpt2)* is RPS2-dependent

**Figure 3:**
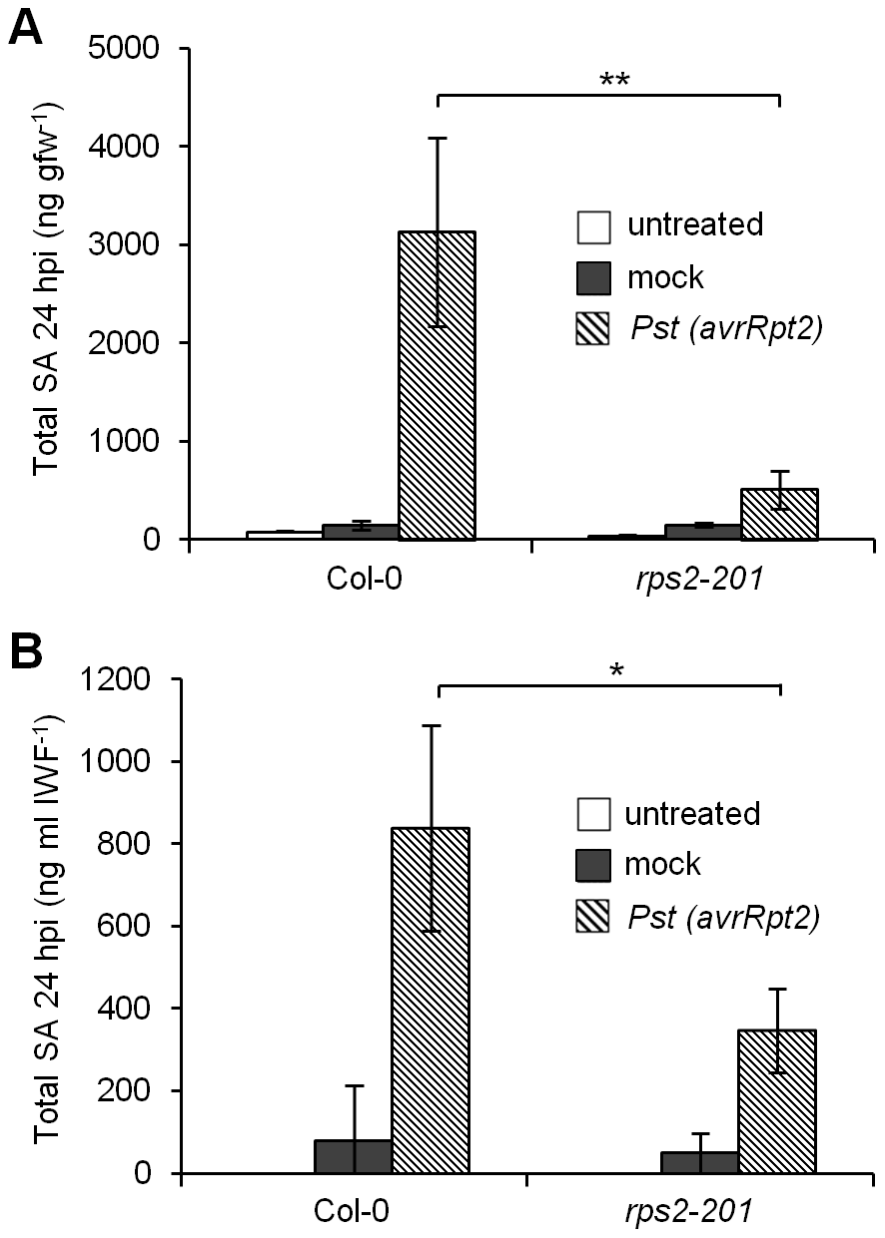
Salicylic acid accumulation in response to *Pst(avrRpt2)* is RPS2-dependent. Intracellular total salicylic acid (SA) levels (A) and intercellular total SA levels (B) were measured at 24 hpi in leaves collected from 4-week-old plants that were untreated, mock-inoculated, or inoculated with 10^6^ cfu ml^−1^ *Pseudomonas syringae* pv. *tomato* carrying *avrRpt2* [*Pst(avrRpt2)*]. The mean ± standard deviation of three replicate samples is shown. Asterisks indicate significant differences between means (T-test, * *P*<0.05, ** *P*<0.01). This experiment was repeated twice with similar results.

SA accumulates (3- to 7-fold, Figure 2) both intra- and intercellularly in young Col-0 during ETI to *Pst(avrRpt2)*. If this response is specific to ETI, SA accumulation should be reduced in the *rps2-201* resistance gene receptor mutant in response to *Pst(avrRpt2)* [13]. Reduced SA accumulation has been observed in whole leaves of *rps2-201* [49], however, inter- and intracellular SA accumulation were not measured. To determine if SA accumulation is specific to the RPS2-AvrRpt2 pathway, intracellular and intercellular SA levels were measured in leaf tissue collected from Col-0 and *rps2-201* mutants that were untreated, mock-inoculated, or inoculated with 10^6^ cfu ml^−1^ *Pst(avrRpt2)* (Figure 3). Untreated and mock-inoculated Col-0 and *rps2-201* leaves contained little intracellular SA (<80 ng gfw^−1^). Col-0 inoculated with *Pst(avrRpt2)* accumulated substantial intracellular SA (3131 ng gfw^−1^) whereas *rps2-201* accumulated 6-fold less (504 ng gfw^−1^). IWFs from untreated and mock-inoculated Col-0 and *rps2-201* contained little SA (<80 ng ml IWF^−1^). IWFs from Col-0 inoculated with *Pst(avrRpt2)* contained high levels of SA (838 ng ml IWF^−1^) whereas IWFs from *rps2-201* contained 2-fold lower SA (346 ng ml IWF^−1^). With respect to both intracellular and intercellular SA levels, *rps2-201* inoculated with *Pst(avrRpt2)* accumulated more SA than mock-inoculated controls but less than Col-0 inoculated with *Pst(avrRpt2)*. Little total SA accumulated in *rps2-201* leaves inoculated with *Pst(avrRpt2)* or in Col-0 inoculated with *Pst* (Figure 2B,D) at 24 hpi, therefore the majority of intracellular and intercellular SA accumulation induced in response to *Pst(avrRpt2)* is dependent on the RPS2-AvrRpt2 pathway.

### Intercellular SA accumulated concurrently with cell death during ETI

**Figure 4:**
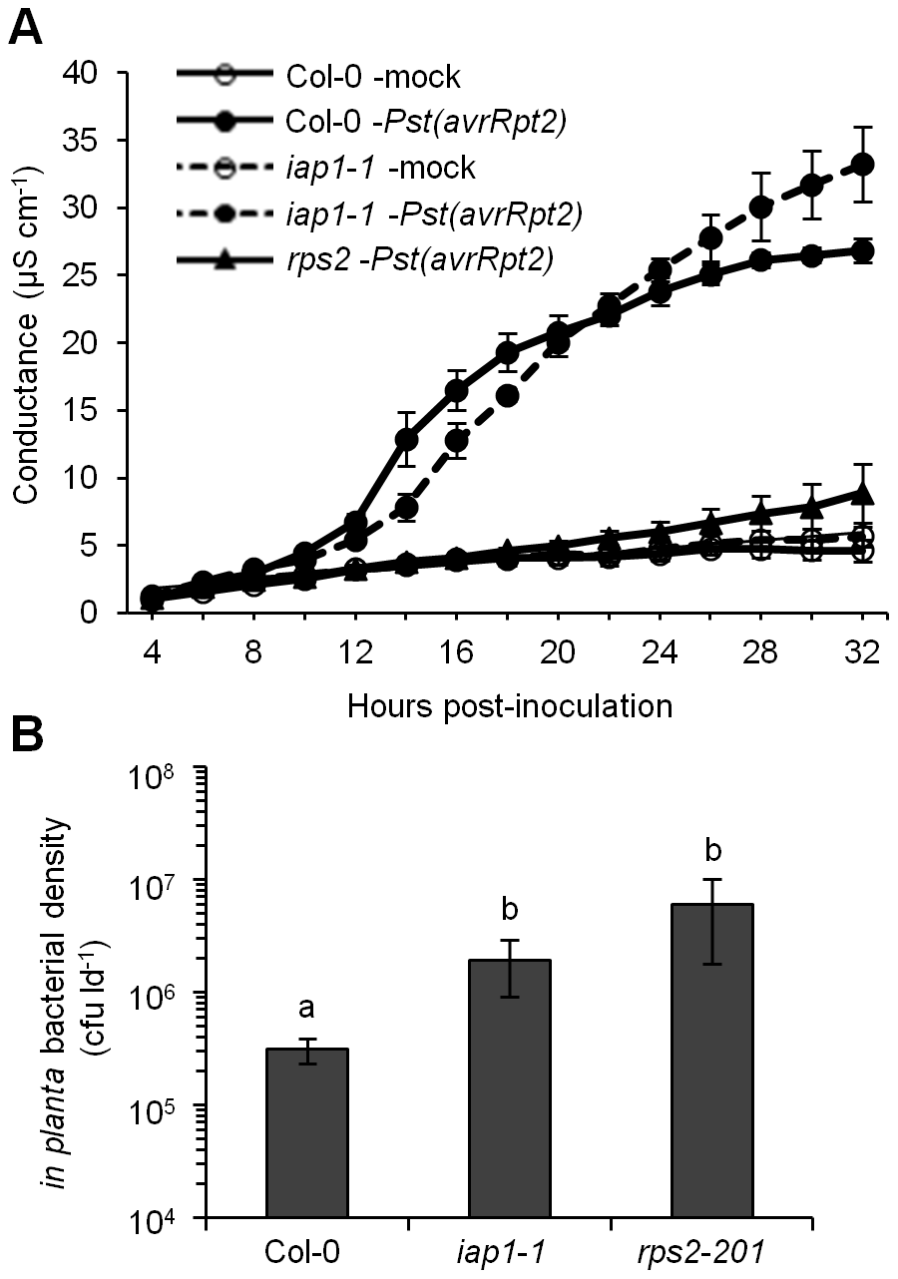
Electrolyte leakage in response to *Pst(avrRpt2)*. (A) Electrolyte leakage was measured in tissue collected from 4-week-old plants inoculated with 10 mM MgCl_2_ (mock) or 10^6^ cfu ml^−1^ *Pseudomonas syringae* pv. *tomato* carrying *avrRpt2* [*Pst(avrRpt2)*]. Values represent the mean ± standard deviation of three sample replicates (B) Four-week-old plants were inoculated with 10^6^ cfu ml^−1^ *Pst(avrRpt2)* and bacterial density (colony-forming units per leaf disc [cfu ld^−1^]) was measured at 3 days post-inoculation. Values represent the mean ± standard deviation of three sample replicates. Different letters indicate significant differences (ANOVA, Tukey's HSD, *P*<0.05). This experiment was repeated once with similar results.

As indicated above, elevated intercellular SA levels were observed by 12 hpi with *Pst(avrRpt2)* in wild-type Col-0 (Figure 2C,D). How SA reaches the intercellular space is not known, however, it is possible that SA leaks from dead or damaged cells during the HR that accompanies ETI. To test this hypothesis, electrolyte leakage was measured in Col-0 and *rps2-201* controls. To make it possible to associate SA accumulation and cell death, plants were inoculated with the same concentration of *Pst(avrRpt2)* that was used for the SA accumulation experiments (10^6^ cfu ml^−1^, Figure 4A). Use of a smaller and more sensitive conductivity meter made it possible to use small, standardized units of leaf tissue (leaf discs), which increased the accuracy of electrolyte leakage measurements. Since a lower bacterial inoculum concentration was used, fewer cells would undergo the HR, therefore cell death was monitored over 32 hours. If cell death occurs before and/or concurrently with SA accumulation, then SA may access the intercellular space from dying and dead cells. Bacterial density measured at 3 dpi demonstrated that *Pst(avrRpt2)* grew to significantly higher levels in *rps2-201* mutants than in Col-0 indicating that an ETI response occurred in Col-0 and was defective in the *rps2-201* mutant (Figure 4B). Electrolyte leakage from Col-0 inoculated with *Pst(avrRpt2)* was significantly greater than in mock-inoculated controls by 10 hpi (T-test, *P*<0.05) suggesting that cell death was occurring by this time (Figure 4A). Thus, the possibility that SA accesses the intercellular space by leaking out of dead cells during ETI could not be ruled out.

Electrolyte leakage was also measured in *iap1-1* to confirm the results of our first set of electrolyte leakage experiments which were carried out with higher inoculum concentrations and a different technique (see materials and methods). *Pst(avrRpt2)* levels supported by *iap1-1* at 3 dpi were significantly higher than in Col-0, consistent with an ETI defect in *iap1-1* (Figure 4B). In this set of experiments electrolyte leakage from *iap1-1* inoculated with *Pst(avrRpt2)* was lower than Col-0 between 14 and 18 hpi, supporting previous evidence for a less robust HR in *iap1-1* relative to Col-0 (Figure 4A). It is interesting to note that electrolyte leakage was greater in *iap1-1* compared to Col-0 from 26 to 32 hpi with *Pst(avrRpt2)*, perhaps due to the fact that *iap1-1* is more susceptible to *Pst* than Col-0, and *Pst* switches to necrotrophy at the end of the infection cycle [50].

### Coronatine suppresses SA accumulation during the compatible *Arabidopsis-Pst* interaction

**Figure 5:**
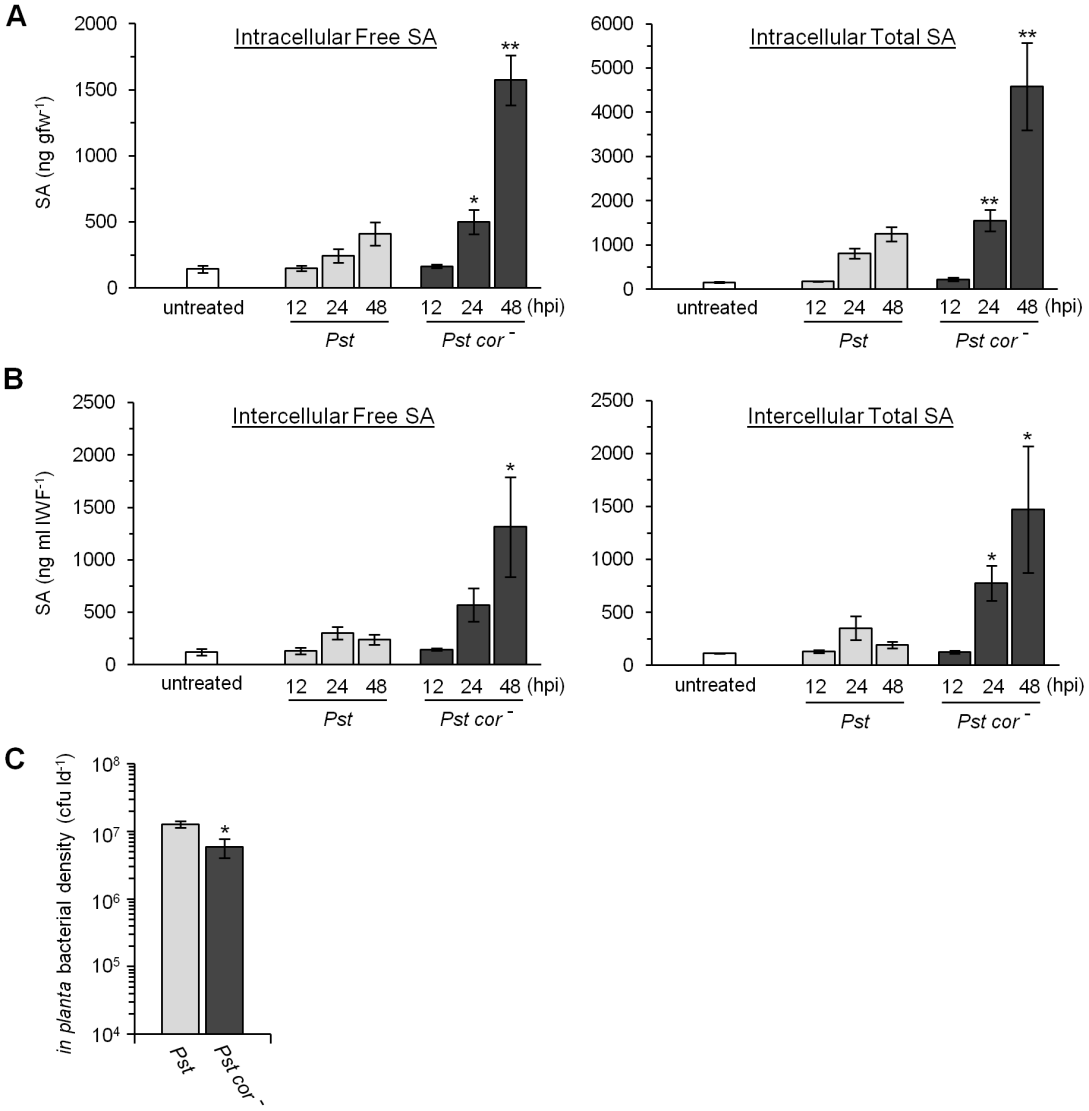
Coronatine suppresses salicylic acid accumulation. Intracellular (A) and intercellular (B) free and total salicylic acid (SA) levels were measured in leaves collected from 4-week-old Col-0 that were untreated or inoculated with 10^6^ cfu ml^−1^ *Pseudomonas syringae* pv. *tomato (Pst)* strain DC3000 or the coronatine-deficient strain DC3661 *(Pst cor^−^)*. The mean ± standard deviation of three replicate samples is shown. Asterisks indicate a significant difference compared to plants inoculated with *Pst* at the corresponding time point (T-test, * *P*<0.05, ** *P*<0.01). (C) Bacterial density (colony-forming units per leaf disc [cfu ld^−1^]) measured at 3 days post-inoculation. Values represent the mean ± standard deviation of three sample replicates. The asterisk indicates a significant difference (T-test *P*<0.01). These experiments were repeated once with similar results.

Since young Col-0 accumulates little intercellular SA during the compatible interaction with *Pst* ([38], Figure 2C,D) we hypothesized that intercellular SA accumulation could be an important part of PTI/basal defense that is suppressed by virulent *Pst*. Work done by other groups demonstrated that the *Pseudomonas* phytotoxin coronatine suppresses SA accumulation in whole leaves [51], [52]. To test whether coronatine suppresses intra- and/or intercellular SA accumulation, young (4 wpg) wild-type Col-0 were inoculated with *Pst* (strain DC3000) or coronatine-deficient *Pst cor^−^* (strain DC3661) followed by intra- and intercellular SA quantification at 12, 24, and 48 hpi (Figure 5A,B). *Pst cor^−^* grew to lower levels than *Pst* by 3 dpi (Figure 5C). As seen previously, Col-0 accumulated modest levels of intracellular free SA by 48 hpi with *Pst* (410 ng gfw^−1^) relative to untreated controls (144 ng gfw^−1^, T-test *P*<0.01). In contrast, higher levels of intracellular free SA accumulated in response to *Pst cor^−^* at 24 and 48 hpi. A similar trend was observed for intracellular total SA levels. Moreover, Col-0 also accumulated higher levels of intercellular SA in response to *Pst cor^−^* relative to *Pst* at 48 hpi (∼1400 ng ml IWF^−1^ compared to ∼200 ng ml IWF^−1^ respectively). These results suggest that *Pst* suppresses both intra- and intercellular SA accumulation in a coronatine-dependent manner during the compatible interaction in young plants.

### Map-based cloning and whole-genome sequencing to identify *iap1-1*

The *iap1-1* mutant was isolated from a screen for ARR-defective plants performed on a population of fast neutron mutants [37]. As demonstrated in this work *iap1-1* is partially compromised in ETI. To identify the causal mutation in *iap1-1* we began with a typical map-based cloning approach. A mapping population was generated and approximately 160 putative homozygous mutant F2s were used to map the *iap1-1* mutation close to marker 461250 (Monsanto Arabidopsis Polymorphism Collection) at 18,087,180 base-pairs (bp) on the long arm of chromosome four. The necessity of screening individual mature plants for an ARR-defective phenotype made the reliable identification of homozygous individuals difficult and time-consuming for fine mapping, therefore we sequenced the *iap1-1* genome in order to locate the mutation.

**Figure 6:**
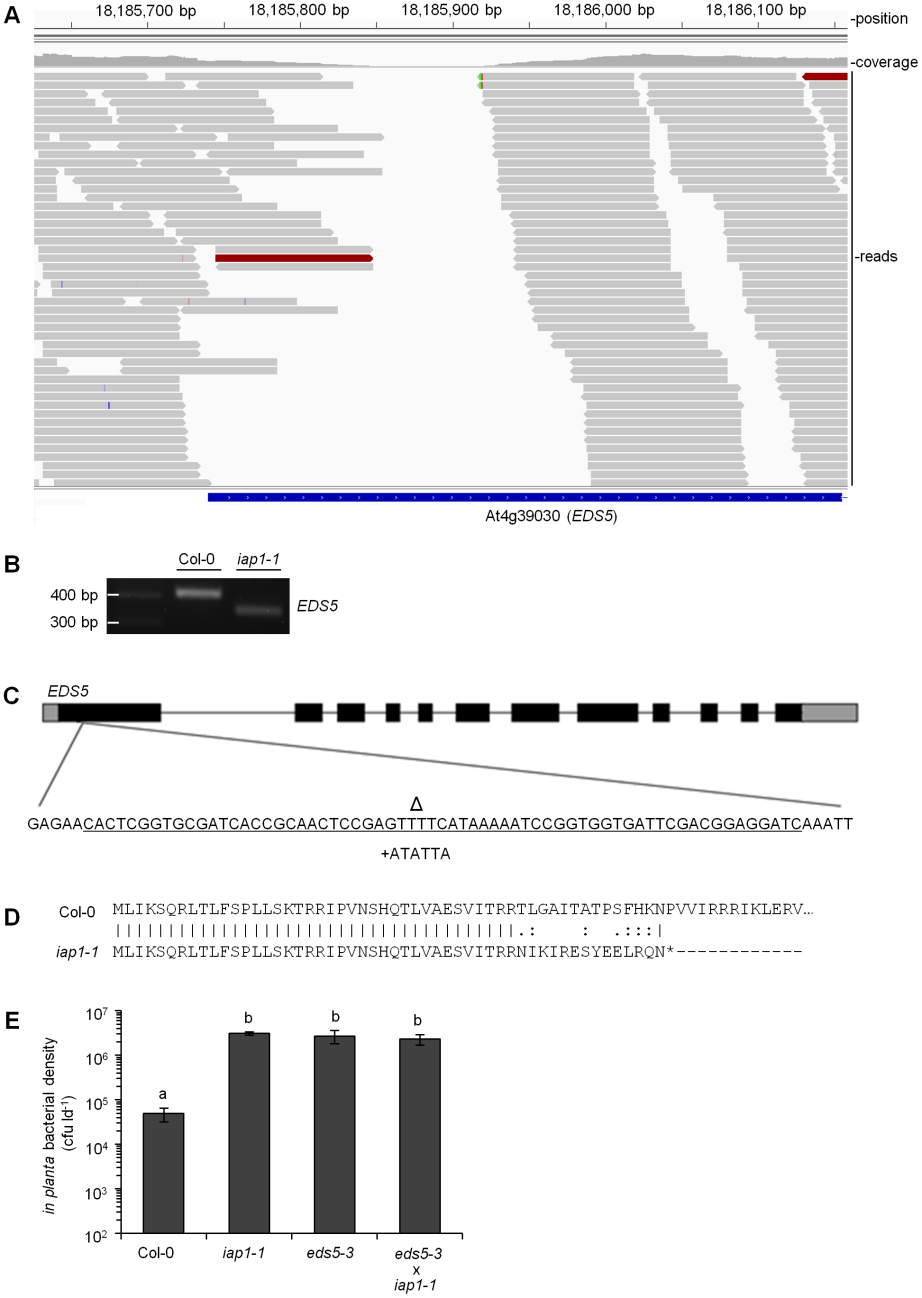
*iap1-1* is an *eds5* mutant. (A) Integrated genome viewer screenshot showing *iap1-1* reads aligned to the Col-0 reference genome. The region of zero-coverage is located in the first exon of *EDS5*. (B) RT-PCR products generated from Col-0 and *iap1-1* using primers that flank the *iap1-1* mutation. (C) *EDS5* gene model including untranslated regions (thick grey lines) exons (thick black lines) and introns (thin black lines). The inset shows the nucleotides that are deleted (marked by Δ and underlined) and inserted (+ATATTA) in *iap1-1* relative to Col-0. (D) Predicted amino acid sequences of Col-0 and *iap1-1* EDS5. A premature stop in the *iap1-1* sequence is indicated by an asterisk. The full Col-0 EDS5 sequence is not shown. (E) Six-week-old Col-0, *iap1-1*, *eds5-3*, and *iap1-1*×*eds5-3* F1s were inoculated with 10^6^ cfu ml^−1^ *Pseudomonas syringae* pv. *tomato* and bacterial density (colony-forming units per leaf disc [cfu ld^−1^]) was determined at 3 days post-inoculation. Values represent the mean ± standard deviation of three sample replicates. Different letters indicate significant differences (ANOVA, Tukey's HSD, *P*<0.05). This experiment was repeated twice with similar results.

DNA isolated from a homozygous *iap1-1* individual was used to create a DNA library that was sequenced using an Illumina HiSeq 1500. This generated roughly 68 million paired-end reads which were then mapped to the Col-0 reference genome (TAIR 10) using BWA software [46] resulting in approximately 50-fold coverage of the genome. Since the majority of fast neutron-generated mutations are deletions [53]–[55] we reasoned that *iap1-1* was likely a deletion mutant. To identify deletions in the *iap1-1* genome we compiled a list of positions in the reference genome that had zero coverage by *iap1-1* reads. A region of zero coverage might indicate that the corresponding sequence is deleted in *iap1-1*. Genome-wide, 814 regions of zero coverage were identified, 244 of which were located on chromosome four. Based on the mapping data, zero-coverage regions located near marker 461250 on chromosome four were investigated. Two zero-coverage regions were found within 500 kb of the marker (Table S1), one of which was located in an intergenic region and was therefore unlikely to represent the causal mutation. Intergenic regions are defined as regions between genes not including recognizable promoters or untranslated regions. The other zero-coverage region was 65 bp within the first exon of *EDS5* (Figure 6A), a gene known to be required for SA accumulation [22] and ARR [19]. Gel electrophoresis of *EDS5* RT-PCR products from *iap1-1* and Col-0 showed a size difference that was consistent with a deletion in the *iap1-1* product (Figure 6B). Sequencing these products confirmed the 65 bp deletion in *iap1-1* and also revealed a 6 bp insertion between the nucleotides flanking the deleted region (Figure 6C). When the Col-0 reference genome was modified to contain this insertion/deletion mutation in *EDS5*, the Illumina reads generated from *iap1-1* mapped continuously across the modified *EDS5* locus, confirming that *iap1-1* harbours the insertion/deletion mutation depicted in Figure 6C. This mutation results in the net loss of 59 nucleotides from the first exon of *EDS5* and causes a frame-shift that produces a premature stop codon after the 52^nd^ amino acid (Figure 6D).

### The *iap1-1* and *eds5-3* mutations are allelic

Both *iap1-1* and *eds5-3* mutants have been shown to be required for ARR and pathogen-induced SA accumulation [19], [22]. To confirm that *iap1-1* is an allele of *EDS5*, homozygous *iap1-1* mutants carrying the *gl1* marker were crossed with *eds5-3* and the F1 progeny were tested for ARR competence at 6 wpg. If the two mutations are not allelic then ARR should be restored in the F1 generation (complementation) but if *iap1-1* and *eds5-3* are allelic then the F1 generation should remain ARR-defective. Wild-type Col-0 supported low *Pst* levels (5×10^4^ cfu ld^−1^) characteristic of ARR whereas *iap1-1*, *eds5-3*, and *iap1-1*×*eds5-3* F1 plants supported high *Pst* levels (at least 50-fold higher than Col-0) indicating that they are compromised for the ARR response (Figure 6E). Therefore, *iap1-1* and *eds5-3* failed to complement each other indicating that these two mutations are allelic and *iap1-1* is a new mutant allele of *EDS5*.

## Discussion

During ARR IAP1 is required for SA accumulation in the intercellular space where SA is thought to act as an antimicrobial agent [37]. This led us to investigate the role of IAP1 and intercellular SA accumulation during ETI. At the same time, map-based cloning, whole-genome sequencing, and complementation analysis identified *iap1-1* as a mutant allele of *EDS5*. In examining the literature, four *eds5* alleles already exist, therefore we propose that *iap1-1* be referred to as *eds5-5* in subsequent publications.

To avoid the time-consuming process of fine mapping, several approaches have been developed that combine the principles of genetic mapping with next-generation sequencing to identify EMS-generated mutations in *Arabidopsis* [56]–[61]. Most of these approaches involve sequencing pools of homozygous mutants selected from an F2 mapping population (reviewed in [62]). This step can be problematic if the mutant phenotype is difficult to score and homozygous individuals cannot be selected reliably. For example, heterozygous *iap1-1/eds5-5* plants display an ARR phenotype that is intermediate to that of homozygous mutants and wild-type plants, and can occasionally be misclassified as homozygous [37]. An alternative approach to avoid this issue is to directly sequence the mutant genome. Unfortunately, EMS mutants usually possess thousands of mutations making it difficult or impossible to identify the causal mutation by direct sequencing alone [62]. This problem can be solved by rough mapping the mutation (to exclude most irrelevant mutations by determining the approximate position of the causal mutation) or backcrossing (to physically remove irrelevant mutations). Ashelford et al. [63] used both techniques followed by whole-genome sequencing to identify the *early bird* (*ebi-1*) EMS mutant. While successful, this effort was complicated by more than 30 non-synonymous mutations that were linked to the causal *ebi1* mutation that were not removed by backcrossing [63]. We used a similar approach combining genetic mapping and whole-genome sequencing to identify the fast neutron mutant *iap1-1/eds5-5*. Ashelford et al. [63] performed four backcrosses to eliminate irrelevant mutations from the *ebi1* mutant, whereas *iap1-1/eds5-5* was backcrossed only twice since the mutation load in fast neutron mutants is typically lower than in EMS mutants [64]–[66]. Therefore, fewer irrelevant mutations will be linked to the causal mutation, making it easier to identify. Based on this information we suggest that identifying fast neutron mutants using a combination of rough mapping and direct sequencing can be done rapidly and efficiently in comparison to EMS mutant identification.

Once the genome sequence data for *iap1-1/eds5-5* was obtained and the reads were aligned to the Col-0 reference genome, identification of potential deletion mutations was straightforward. Instead of generating a list of SNPs as would be done for an EMS-generated mutant, we compiled and assessed regions of the reference genome that were not covered by reads generated from the *iap1-1/eds5-5* mutant genome. A region of zero coverage could indicate that the corresponding sequence is deleted or highly polymorphic in *iap1-1/eds5-5* or could represent an error in the reference genome [67]. Regions of low coverage also occur for other reasons, including the difficulty of sequencing some genomic regions (e.g., low GC areas). Since we did not sequence the wild-type parent of *iap1-1/eds5-5* we cannot differentiate between these possibilities. Therefore, while 814 regions of zero coverage were identified, this is probably an overestimate of the fast neutron-induced mutations in *iap1-1/eds5-5*. Fast neutron mutant genomes are estimated to harbour deletions in approximately 10 genes [66]. Our bioinformatics strategy did not detect the co-localized insertion mutation present in *iap1-1/eds5-5*. The insertion was discovered later by Sanger sequencing demonstrating the necessity of confirming the molecular basis of fast neutron-generated mutations. Although not as common as deletions, other instances of co-localized insertion-deletion mutations resulting from fast neutron mutagenesis have been described [68], [69].

To identify IAP1, we began by genetically mapping it to chromosome four and turned to whole-genome sequencing because it became available and additional mapping became unfeasible. Based on this experience, we propose a more rapid approach for gene identification in which rough mapping and sequencing are performed at the same time. As soon as the causal mutation is mapped to one chromosomal region, the sequencing data can be used to obtain a list of zero-coverage regions in this area. Zero-coverage regions in introns or intergenic sequences can be excluded as they are unlikely to affect gene function. The list of candidate zero-coverage regions in exons, promoters, and untranslated regions would be assessed to determine if mutations in any of these genes makes sense based on the mutant phenotype. If there are too many zero-coverage regions (>10), then further mapping may be required to reduce the number of candidate genes. If as expected for fast neutron-induced mutants, there are only a few zero-coverage regions in candidate genes, complementation analysis can be performed to confirm the identity of the gene. If whole-genome/next-generation sequencing had been available when we started to map *iap1-1/eds5-5*, we would have mapped it near one or two markers on chromosome four, then realized that there was just one zero-coverage region in one gene near these markers making us confident that this was the mutation responsible for the *iap1-1/eds5-5* phenotype. Moreover, this gene encoded EDS5, a SA transporter important for defense and therefore made sense with respect to the ARR-defective phenotype of *iap1-1/eds5-5*. Many years of challenging mapping would have been avoided if this method (simultaneous mapping and whole-genome sequencing) had been available.

Plants that are heterozygous for the *iap1-1/eds5-5* mutation display an ARR phenotype that is intermediate to that of homozygous mutants and wild-type plants, suggesting that *iap1-1/eds5-5* is a semidominant mutation [37]. Other mutant alleles of *eds5* have been classified as recessive (*eds5-1* [30] and *eds5-3* [22]). We believe this difference reflects the nature of assessing dominance in disease resistance mutants. For example, small changes in humidity may affect the growth of *Pst* in plants [70] such that heterozygotes may exhibit phenotypes that are similar to homozygous mutants or wild-type plants. Therefore during heterozygote analysis, if enough heterozygotes are classified with a wild-type phenotype, a semidominant mutation will be classified as recessive. It is also possible that *iap1-1/eds5-5* is a unique semidominant allele. Since *EDS5* transcripts are detectable in *iap1-1/eds5-5* mutants, a truncated EDS5 protein may be produced in *iap1-1/eds5-5* as 52 amino acids precede the premature stop codon in the *iap1-1/eds5-5* mutant. If the mutant peptide has a dominant negative effect on the wild-type protein this could explain the semidominant phenotype observed in heterozygous *iap1-1/eds5-5*. Other *eds5* mutant alleles that have been studied have been classified as recessive and also have premature stop codons and may produce truncated proteins [27]. However, the stop codons in *eds5-1, eds5-3*, and *iap1-1*/*eds5-5* occur at different positions suggesting that distinct peptides could be produced. Future studies to investigate whether these mutant alleles produce different peptides may shed light on the importance of the functional domains of the EDS5 MATE transporter. It is also possible that the semidominant phenotype of *iap1-1/eds5-5* is the result of an additional, unknown mutation.

We do not usually observe a difference in disease susceptibility between young Col-0 and *iap1-1/eds5-5* during the compatible interaction with virulent *Pst* [37, this study]. In some of our experiments young *iap1-1* is slightly more susceptible than Col-0, however, the difference is not always statistically significant. This observation conflicts with other studies of *eds5* mutants [22], [30], [31]. We propose that this difference may result from our use of a 10-fold higher inoculum concentration (10^6^ cfu ml^−1^) during bacterial growth assays. Indeed, Glazebrook et al. [30] indicate that the enhanced disease susceptibility phenotype of *eds* mutants is more easily observed when lower inoculum concentrations are used.

Although *IAP1* is *EDS5*, a gene already known to be required for SA-mediated defense responses, both confirmatory and novel results were obtained during our investigation of compatible and incompatible (ETI) responses to *P. syringae* in *iap1-1/eds5-5*. Bacterial levels were modestly enhanced in *iap1-1/eds5-5* compared to Col-0 in response to both *Pst(avrRpt2)* and *Pst(avrRps4)* indicating that the ETI response was reduced, but not abolished in *iap1-1/eds5-5*, suggesting that IAP1/EDS5 contributes to ETI. Similar results were also observed in *eds5-3/sid1* in response to *Pst(avrRpt2)* [22]. In addition, Venugopal and colleagues [35] found that SA-deficient *sid2* mutants are also partially compromised for ETI. ETI was fully compromised in *sid2 eds1* double mutants suggesting that SA and EDS1 function redundantly during ETI. A macroscopic HR was observed in *NahG*, other *eds5* alleles [8], [32], and *iap1-1/eds5-5*, and trypan blue staining detected similar levels of cell death in *iap1-1/eds5-5* and Col-0 (this study). Neither of these techniques is sensitive or quantitative, therefore electrolyte leakage assays were used to carefully examine the HR in *iap1-1/eds5-5*. Electrolyte leakage was modestly reduced in *iap1-1/eds5-5* compared to Col-0 inoculated with 10^6^ or 10^7^ cfu ml^1^ *Pst(avrRpt2)* at several time points. Like the trypan blue assays, the electrolyte leakage assays confirm that HR cell death occurs in *iap1-1/eds5-5*, however, this sensitive assay indicates that the HR response was modestly reduced. We confirm that IAP1/EDS5 contributes to bacterial growth restriction during ETI and demonstrate that the HR is also partially dependent on functional IAP1/EDS5. In other words, it appears that IAP1/EDS5-dependent SA accumulation is required for a full HR and the bacterial growth restriction that takes place during RPS2-AvrRpt2-mediated ETI. In addition, electrolyte leakage assays revealed that cell death and intercellular SA accumulation occurred concurrently, suggesting that SA may gain access to the intercellular space from dead and dying cells during the HR. Consistent with this idea, the highest levels of intercellular SA accumulation were typically observed at 24 or 48 hpi with *Pst(avrRpt2)*, once extensive cell death had occurred. The timing of maximal intercellular SA accumulation varied between experiments, potentially because the timing and strength of the HR can be affected by variations in humidity that occur even within growth chambers.

SA accumulates in the intercellular space during ARR where it is thought to function as an antimicrobial agent. The idea that SA might act as an antimicrobial agent in the intercellular space in other defense responses has not been examined, therefore we measured both inter- and intracellular SA accumulation during ETI initiated by *Pst(avrRpt2)* and in response to virulent *Pst* (compatible interaction). Wild-type Col-0 accumulated intercellular SA in response to *Pst(AvrRpt2)* in the same range as observed during ARR (153 to 400 ng ml IWF^−1^) [37], [38] providing evidence for the importance of intercellular SA accumulation during ETI. Additionally, SA accumulation was reduced in *rps2-201* mutants indicating that the intercellular SA produced was specific to the RPS2-AvrRpt2 ETI pathway. In comparison, intercellular SA levels in *iap1-1/eds5-5* inoculated with *Pst(avrRpt2)* were similar to background levels observed in untreated and mock-inoculated controls demonstrating that *iap1-1/eds5-5* accumulates little SA during ETI as would be expected of an *eds5* mutant [22]. Although intercellular SA accumulation was reduced to background levels in *iap1-1/eds5-5*, RPS2-AvrRpt2-initiated ETI modestly reduced bacterial levels, suggesting that inter- and intracellular SA accumulation, as well as SA-independent constituents contribute to ETI.

During the response to virulent *Pst* over 48 hpi, *iap1-1/eds5-5* accumulated low levels of inter- (free and total) and intracellular (free) SA, similar to untreated or mock-inoculated plants, demonstrating that both intra- and intercellular SA accumulation is reduced by the *iap1-1/ed5-5* mutation. We also observed that total intracellular SA accumulation in Col-0 and *iap1-1* was low at early times after inoculation with virulent *Pst* (12 and 24 hpi), however, by 48 hpi, total intracellular SA levels increased to ∼1000 ng gfw^−1^ in *iap1-1/eds5-5* and to ∼1800 ng gfw^−1^ in Col-0, similar to SA levels induced by *Pst(avrRpt2)*. In Col-0 responding to virulent *Pst*, both intracellular and intercellular free SA levels were similar to background levels (untreated or mock-inoculated plants) with the exception of a modest increase in intracellular free SA levels at 48 hpi. One explanation for these observations is that PTI-associated SA accumulation is suppressed by virulent *Pst*. Support for this hypothesis comes from two studies in which the *Pseudomonas* phytotoxin coronatine was shown to inhibit whole-leaf SA accumulation in *Arabidopsis* in response to virulent *Pst* and *Ps maculicola* [51], [52]. In addition, SA accumulates during PTI/basal resistance in response to the PAMP flg22 [36]. These investigations suggest that coronatine is important in suppressing SA accumulation during the PTI response and this produces a compatible environment for *Pseudomonas* infection. In this study we demonstrated that coronatine-producing *Pst* suppress both intracellular and intercellular SA accumulation. These data support the idea that intercellular SA accumulation is an important component of the PTI response.

Serrano et al. [28] speculate that *eds5* mutants do not accumulate SA because the EDS5 MATE transporter is not functional such that SA remains trapped in the chloroplast leading to high SA levels and feedback inhibition of SA biosynthesis. It is interesting to note that at the later 48 hpi time point, *iap1-1/eds5-5* accumulated elevated levels of total intracellular SA (free+conjugated) in response to both virulent and avirulent *Pst*. Since little intracellular free SA accumulated at 48 hpi, the conjugated form of SA must be accumulating. We speculate that free SA is quickly converted to its conjugated form at 48 hpi with *Pst* and therefore little free SA is present for feedback inhibition of SA biosynthesis, allowing intracellular conjugated SA levels to rise. Also of note, both *iap1-1/eds5-5* and Col-0 were similarly susceptible to *Pst* even though *iap1-1/eds5-5* accumulated ∼500 ng gfw^−1^ less intracellular total SA at 48 hpi compared to Col-0. Although free SA is generally regarded as the active form, studies on mutants in which enhanced disease susceptibility corresponds primarily with a reduced capacity to accumulate conjugated SA suggest that conjugated SA could be an important part of a successful defense response [71]–[74]. However, we speculate that by 48 hpi, suppression of PTI defense by *Pst* is waning such that intracellular conjugated SA accumulates in wild-type Col-0, however it is too late to mount a successful defense as bacteria have multiplied to high levels. It is also possible that the absence of accumulation of free SA in the intercellular space is responsible for the unsuccessful defense response in Col-0 and *iap1-1/eds5-5* to virulent *Pst*.

## Conclusions

By developing a whole-genome/next-generation sequencing technique to identify deletion mutations, we identified a new mutant allele of *EDS5*. This technique will be useful for other researchers as it allows rapid identification of a deletion mutant by whole-genome sequencing once the mutation is roughly mapped. Our studies of *iap1-1/eds5-5* revealed that SA accumulates in both the inter- and intracellular spaces during the RPS2-AvrRpt2-initiated ETI response. This suggests that SA contributes as both an intracellular signaling molecule and an antimicrobial agent in the intercellular space. We also demonstrated that intercellular SA accumulation is suppressed in a coronatine-dependent manner by virulent *Pst*. Therefore, SA may act as an antimicrobial agent in the intercellular space during PTI/basal resistance in *Arabidopsis*.

## Materials and Methods

### Plant growth conditions

The *iap1-1* [37], *rps2-201* [13], and *eds5-3/sid1* [22] mutants were used in conjunction with wild-type Columbia (Col-0). *iap1-1* was isolated from 5000 M2 fast neutron-mutagenized seeds (Lehle Seeds, Texas, USA) [37]. Seeds were surface-sterilized in 70% ethanol for 2 minutes and in sterilization solution (1.6% bleach, 0.1% Tween 20) for 10 minutes then washed with sterile water 5 times. Seeds were stratified at 4°C for at least 2 days before they were left to germinate on Murashige and Skoog (1962) medium for 5–7 days under continuous light. Seedlings were transferred to soil at the cotyledon stage (Sunshine Mix No. 1 [JVK]) and watered with 1 g L^−1^ 20-20-20 fertilizer. Temperature was maintained between 22°C and 24°C during a 9-hour photoperiod with an average light intensity of 150 µE m^−2^·sec^−1^. Humidity ranged between 75% (winter) and 85% (summer).

### Disease resistance assays

Pseudomonas syringae pv. tomato (Pst) strain DC3000 (rifampicin and kanamycin resistant) and Pst(avrRpt2) were obtained from Dr. A. Bent (University of Wisconsin at Madison; [39]). Pst(avrRps4) was obtained from Dr. R. Subramaniam (Agriculture Canada). Pst cor^−^ (strain DC3661) was obtained from Dr. D. Cuppels (Agriculture Canada; [40]). Bacteria were shaken overnight at room temperature (22–24°C) in King's B media and kanamycin (50 µg ml^−1^) to mid-log phase then resuspended in 10 mM MgCl_2_ at a concentration of 10^6^ or 10^7^ cfu ml^−1^. Plants were inoculated by pressure infiltration and in planta bacterial levels were determined as previously described [19], [41].

### Trypan staining and electrolyte leakage assays

Plants were inoculated with *Pst(avrRpt2)* (10^7^ or 10^6^ cfu ml^−1^) or mock-inoculated with 10 mM MgCl_2_. For trypan staining leaves were harvested at 24 hours post-inoculation (hpi) and immediately submersed in staining solution (0.02 g trypan blue, 8% phenol, 8% glycerol, 8% lactic acid, 8% water, 67% 95% ethanol). Leaves were boiled for 1 minute in the staining solution and left overnight followed by destaining in 70% ethanol. Images were captured using a digital camera (Nikon DXM1200F) mounted on a Nikon eclipse TE2000-S microscope at 10× magnification. For the first set of electrolyte leakage assays (Figure 1C) leaves were collected at 2 hpi, weighed, and rinsed in distilled water. 48 leaves per treatment were added to 80 ml of distilled water in triplicate and conductance was measured using a YSI environmental model 556 conductivity meter. For the second set of electrolyte leakage assays (Figure 4A) leaf discs were collected at 1 hpi, rinsed in nanopure water for 1 hour, then transferred to vials containing 10 ml nanopure water (9 plants per treatment, 10 leaf discs per vial, 3 vials per treatment). Conductance was measured periodically using a Jenway model 4070 portable conductivity meter.

### IWF collection and SA measurement

IWF (intercellular washing fluid) collections were performed as described previously [19], [38]. Briefly, Col-0 and *iap1-1* (4 weeks-old) were untreated, mock-inoculated (10 mM MgCl_2_) or inoculated with 10^6^ cfu ml^−1^ virulent *Pst*, avirulent *Pst(avrRpt2)*, or coronatine-deficient *Pst*. Leaves were harvested at 12, 24, and 48 hpi then surface-sterilized (50% ethanol and 0.05% bleach) and vacuum infiltrated with sterile water. Leaves were then blotted dry and centrifuged at 1000×g for 30 minutes in 50 ml syringes fitted into microcentrifuge tubes. The IWFs were filter-sterilized to remove *Pst*. ADPWH_*lux* is a non-pathogenic soil bacterium that has been modified to produce luciferase proportional to the amount of SA present [42] and was used to measure free SA and total SA (free SA plus SA-glucosides) as described previously [43]. Briefly, ADPWH_*lux* was grown overnight in LB media with shaking at 37°C and then diluted to an OD_600_ of 0.4 with fresh LB followed by incubation with IWFs or leaf tissue minus IWFs for 1 hour at 37°C in an opaque 96-well plate (Corning no.:3915). Luminescence was measured on a Biotek plate reader at 490 nm and used to calculate SA concentrations.

### Map-based cloning

*iap1-1* (Col-0 background) was crossed to Landsberg erecta (Ler) and the cross was confirmed in the F1 generation using InDel molecular markers [44]. Screening for homozygous *iap1-1 or IAP1* in the F2 generation was done by inoculating mature plants with virulent *Pst* (10^6^ cfu ml^−1^) and determining *in planta* bacterial levels after 3 days. Plants were classified ARR-incompetent if bacterial levels were ≥10^7^ cfu ld^−1^ and ARR-competent if bacterial levels were ≤10^5^ cfu ld^−1^. InDel and CAPS molecular markers [44] were used to map the *iap1-1* mutation to the long arm of chromosome four [45]. Primers for significantly linked markers include AGAGGCAAGTGAATCAACCGT, ACTTCGCAGCTCTCTGTTTTG (461250); GGGCAAGGTAATTGATCGTCT, GCTTCTATAATCCGTGATCCG (461266); and GCACAAGGGAGAGAGATTCAA, GGATTCGAGAATTTCGGCA (460755). Primers for CAPS markers include GGAGATTGTTGAATACACTTGCTAC, GGCTAAGTTATGCAATATATTTCTCTT (18,268,815 bp); and TGCCGAGAAAATCAGAGACA, CGACCTGAAAGACGTTAAATCC (18,111,371 bp) digested with PstI and RsaI, respectively.

### Whole-genome sequencing

DNA was isolated from leaf tissue of an *iap1-1* plant using a modified phenol-chloroform extraction method. Briefly, tissue was ground in liquid nitrogen, incubated with DNA extraction buffer (200 mM Tris-HCL [pH 7.5], 250 mM NaCl, 25 mM EDTA, 0.5% SDS [w/v]), extracted with phenol-chloroform, and precipitated in isopropanol. The pellet was re-suspended and treated with RNase Cocktail (Life Technologies) followed by extraction with phenol-chloroform and again with chloroform alone. The DNA was then precipitated in ethanol. Library preparation was carried out according to manufacturer instructions (Nextera) and sequencing was performed on an Illumina HiSeq 1500 (high output mode) using approximately one third of a lane. Reads were aligned to the *Arabidopsis* Col-0 reference genome (TAIR 10) using BWA software [46].

### RT-PCR

Leaf tissue was harvested, flash-frozen in liquid nitrogen, and stored at −80°C. RNA was isolated using Sigma TRI Reagent according to the manufacturer's instructions. Residual DNA was degraded using TURBO DNA-free (Life Technologies) prior to RNA quantification. First-strand cDNA synthesis was carried out using M-MLV Reverse Transcriptase (Life Technologies). PCR primers used to amplify the *iap1-1* polymorphism were: 5′- ATGCTAATCAAATCCCAAAGATTGAC (F) and 5′- CAGCGAGTTCGATGGAGC (R) at 29 cycles. Products were visualized on a 2% agarose gel stained with ethidium bromide.

## Supporting Information

Figure S1 **Trypan blue staining for hypersensitive response cell death.** Four-week-old Col-0 and *iap1-1* were inoculated with 10 mM MgCl_2_ (mock) or 10^7^ cfu ml^−1^ *Pseudomonas syringae* pv. *tomato (Pst)* carrying *avrRpt2*. Leaves were stained with trypan blue at 24 hours post-inoculation.
(TIF)

Table S1 **Positions on the Col-0 reference genome near marker 461250 (18,087,180 bp) that were not covered by** ***iap1-1*** **Illumina sequencing reads.**
(PDF)
