# Supplementary figures and images for: Investigation of Intercellular Salicylic Acid Accumulation during Compatible and Incompatible Arabidopsis-Pseudomonas syringae Interactions Using a Fast Neutron-Generated Mutant Allele of EDS5 Identified by Genetic Mapping and Whole-Genome Sequencing
Source: PLoS One. 2014 Mar 4;9(3):e88608. doi: 10.1371/journal.pone.0088608 (PMC3942312; doi:10.1371/journal.pone.0088608)

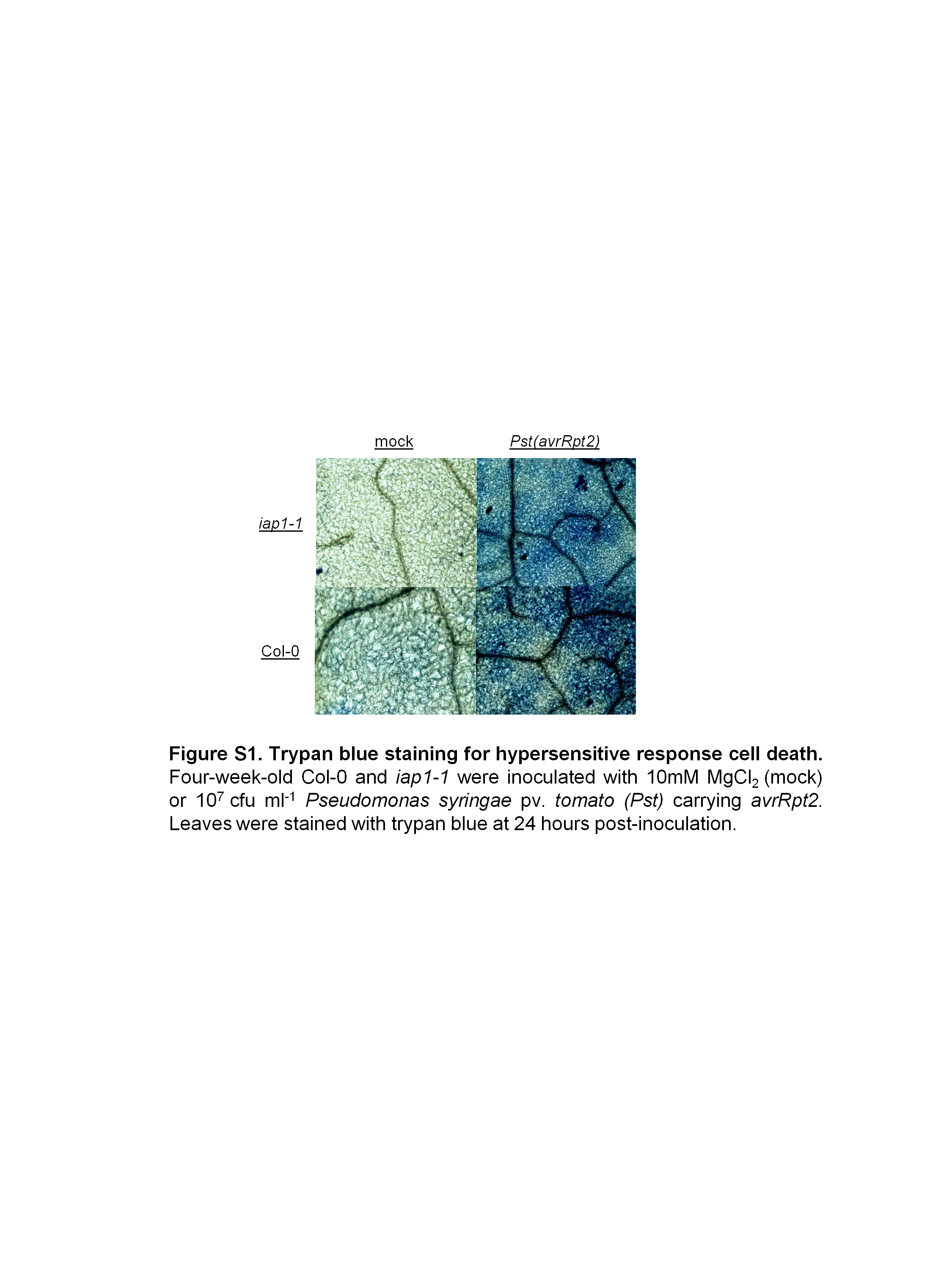

Supplement: Figure S1 — Trypan blue staining for hypersensitive response cell death. Four-week-old Col-0 and iap1-1 were inoculated with 10 mM MgCl2 (mock) or 107 cfu ml−1 Pseudomonas syringae pv. tomato (Pst) carrying avrRpt2. Leaves were stained with trypan blue at 24 hours post-inoculation. (TIF) [file pone.0088608.s001.tif]
