# Supplementary material for: Investigation of Intercellular Salicylic Acid Accumulation during Compatible and Incompatible Arabidopsis-Pseudomonas syringae Interactions Using a Fast Neutron-Generated Mutant Allele of EDS5 Identified by Genetic Mapping and Whole-Genome Sequencing
Source: PLoS One. 2014 Mar 4;9(3):e88608. doi: 10.1371/journal.pone.0088608 (PMC3942312; doi:10.1371/journal.pone.0088608)
Supplement: Table S1 — Positions on the Col-0 reference genome near marker 461250 (18,087,180 bp) that were not covered by iap1-1 Illumina sequencing reads. (PDF) [file pone.0088608.s002.pdf]

Table S1. Positions on the Col-0 reference genome near marker 461250 (18,087,180 bp) that were not covered by *iap1-1* Illumina sequencing reads.

**Position (chromosome 4)**	**Size (bp)**	**Region**	**Description**
18,585,053 - 18,585,056	4	intergenic	
18,185,855 - 18,185,919	65	exon	At4G39030 (*EDS5)*
17,405,532	1	intergenic	
17,362,499 - 17,362,513	15	intergenic	
17,357,652	1	intergenic	
17,357,246 - 17,357,356	12	intergenic	
17,317,390 - 17,317,401	12	intergenic	
17,317,380	1	intergenic	
17,315,542	1	intron	AT4G36740
17,315,483 - 17,315,494	12	intron	AT4G36740
17,313,889 - 17,313,894	6	intergenic	
17,276,554 - 17,276,587	34	5’ UTR	AT4G36630
17,261,784 - 17,261,803	20	intron	AT4G36590
